# The temporalities of policymaking: The case of HIV test-and-treat policy adoption in Zimbabwe

**DOI:** 10.1016/j.healthplace.2019.102246

**Published:** 2020-01

**Authors:** Meg Moran, Morten Skovdal, Edith Mpandaguta, Rufurwokuda Maswera, Noah Kadzura, Freedom Dzamatira, Constance Nyamukapa, Simon Gregson, Malebogo Tlhajoane

**Affiliations:** a Department of Public Health, University of Copenhagen, Copenhagen, Denmark; b Biomedical Research and Training Institute, Harare, Zimbabwe; c School of Public Health, Imperial College London, London, UK

**Keywords:** Policy studies, Temporality, Test-and-treat, HIV, Zimbabwe

## Abstract

Despite calls for “rapid adoption” of global health policies and treatment guidelines; there is little understanding of the factors that help accelerate their adoption and implementation. Drawing on in-depth interviews with sixteen Zimbabwean policymakers, we unpack how different factors, rhythmic experiences and epochal practices come together to shape the speeding up and slowing down of *test-and-treat* implementation in Zimbabwe. We present an empirically derived framework for the temporal analysis of policy adoption and argue that such analysis can help highlight the multiple and messy realities of policy adoption and implementation - supporting future calls for ‘rapid’ policy adoption.

## Highlights

• There are calls for the “rapid adoption” of global health policies.
• There is a need to understand the temporalities of policy adoption and implementation.
• We unpack the assemblage of temporalities found to speed up, or slow down, HIV test-and-treat policy adoption in Zimbabwe.
• We present a framework for the temporal analysis of policy adoption.

## 1 Introduction

Despite growing interest in ‘rapid’, ‘timely’, ‘speedy’, ‘fast tracked’ policy adoption and implementation in the global health field, there has been little conceptualisation of how temporality features in health policy adoption analyses in low- and middle-income countries (Gilson and Raphaely, 2008; Walt et al., 2008). In this article, we show how temporality becomes heightened in the context of global calls for ‘rapid’ policy adoption and implementation and develop an empirically derived framework for the temporal analysis of policy adoption and implementation.

While there is an increasingly good understanding of the factors that determine policy adoption and implementation (Fischer et al., 2015; Sabatier, 1999; Yanow, 1996), there is less understanding of the factors that help *accelerate* policy adoption and implementation. This is arguably due to the dominance of the more ‘conventional’ policy transfer logic, which frames much policy as taken-for-granted expressions of the issuing authority's knowledge to be ‘delivered’ in discrete forms via local administrations, officials, practitioners or citizens (Jenkins, 2007). This logic inhibits research into the temporality of policy adoption by assuming linearity, where time is a discrete continuum and ‘history’ is seen as a single entity of sequenced events. The starting point of this investigation is based upon the premise that to understand how a policy comes to be “rapidly” adopted and implemented, we must explore the “messiness” and tension between the smooth spaces imagined by the global policy models and policymakers, and the mundane and messy reality of day-to-day delivery (Campbell et al., 2012a, 2012b; Kingfisher, 2013; Sheller and Urry, 2006). Against this background, we present a case study of the journey of *test-and-treat* in Zimbabwe to examine the factors accelerating or slowing down the roll-out of this global HIV policy. We examine the moments of rupture, transition and transformation of *test-and-treat* as it moves, gets adopted and implemented (Cresswell, 2010; Massey, 2011; McCann and Ward, 2013) - paying particular attention to the place-specific factors, collective experiences, socially organised activities and practices that affect the temporalities of *test-and-treat* policy adoption.

### 1.1 Locating ‘temporality’ in the critical policy literature

We live in an increasingly interconnected world, where ideas, people and technologies seem to be moving globally at incredible speed. New policy ideas are among the travelling technologies to speed across borders as best-practice models; providing fertile ground for ‘a new generation of social constructivists’ to pay attention to the temporal and spatial dynamics of policymaking activities (Peck and Theodore, 2015).

Policy literature, which assumes policies are developed at one time or place, to be learnt, adapted and adopted for another time and place, often represents the logic of ‘policy transfer’ (Dolowitz and Marsh, 2000). According to this approach, policies are seen as discrete products capable of being ‘transferred’ from one setting to the next (Clarke et al., 2015). Policies are often conceptualised as discrete, fixed and unidirectional products (Clarke et al., 2015), which diffuse outwardly and unidirectionally from a single place of intervention to be ‘delivered’ in discrete forms via local administrations, officials, practitioners or citizens (Jenkins, 2007). In these representations, it is often presumed that implementation and compliance will follow (Clarke et al., 2015). Conceptualising policies in this way represents a certain logic of rationality which pervades in “conventional” policy analysis (Clarke et al., 2015). This “conventional” logic is often found in policy analyses within literature on public administration, political science, organisational theory, public management research (Pülzl and Treib, 2006).

Against the background of the ‘traditional’ and ‘conventional’ theories of policy analysis is a *critical perspective,* which draws attention to what Howard (2005:3) refers to as the “complex, value-laden nature of the policy process, as well as the primary role of political power in determining the direction of public policy”. Distinct fields of interpretive policy analysis (IPA) and critical policy studies (CPS) challenge the orthodoxy of the conventional approaches by offering alternative explanations and conceptualisations for how policy is implemented (See for example Clarke et al., 2015; Fischer et al., 2015; Hill and Hupe, 2002; Lendvai and Stubbs, 2007; Yanow, 1999, 2004). These explanations often focus on the nature of social problems, the design of the policy, the governance system and organisational arrangements in which the policy operates, and the will or capacity of the people charged with implementing the policy (Spillane et al., 2002). In recent years, there have been trends within CPS, and the wider social science field, to highlight the “messiness” of policy movement by exploring what happens to policies as they move through various sites of policy delivery and reception (Kingfisher, 2013). The focus of investigation becomes the moments of rupture, transition and transformation of policy as it moves (Cresswell, 2010; Massey, 2011; McCann and Ward, 2011). According to such approaches, policies undergo dynamic processes of movement and ‘mutation’; they are not merely being *transferred,* but “their form and their effects are transformed by these journeys” (Peck, 2011:793). Geography scholars have directly sought to address some of the conceptual shortcomings of policy transfer literature by foregrounding an interest in the ‘spatio-temporal’ context in which a policy moves; under an investigation of ‘policy mobilities’ (McCann and Ward, 2011; Peck, 2011; Peck and Theodore, 2010; Temenos and McCann, 2013). However, according to Wood (2015: 568) the policy mobilities literature does not go far enough to consider the temporal elements comprised of policy adoption and implementation, beyond the “frantic and fast”. While Wood, in response, offers uselful insights into policy slowness, demonstrating the gradual, repetitive and delayed aspects of policy circulation and adoption, she suggests a chronological and historical approach, which we believe falls short of seeing the iterative and dynamic interaction between history, the present and expectations for the future. To further advance the temporality of policy mobilities, we draw on Cresswell's (2010) seminal work on the politics of mobility, which discusses the constituent parts of mobility, including the speed or slowness of how a thing (in our case, a policy) moves. Reflecting our interests, he considers why something moves, how fast something moves, what route it takes and how it stops. We also take inspiration from Adam's (2008: 4) work on ‘timescapes’, which considers temporality in relation to spatiality, materiality and contextuality, both to study the dynamics of action and to explore how “the socio-environmental world is formed, maintained and reworked over time.” We draw on all of these perspectives to explore and conceptualise the temporal factors, experiences and practices that interact in iterative and dynamic ways to affect the speed and/or slowness of policy adoption and implementation.

### 1.2 The HIV *test-and-treat* policy

Global health organisations are among those who frequently update their guidelines in response to emerging evidence and set global disease-specific targets with the goal of eliciting rapid adoption at the national level (Gilson and Raphaely, 2008; Gupta and Granich, 2016). Clinical guidelines for HIV testing and treatment are no different and have undergone several alterations over the years. For instance, in November 2015 the World Health Organisation (WHO) published guidelines recommending expanding treatment eligibility for anyone who is HIV-infected, regardless of their immunological status (WHO clinical stage or CD4 count). This *treat all* paradigm, which includes a policy recommendation to *test-and-treat*, removes all limitations on antiretroviral therapy (ART) among people living with HIV (PLHIV) (WHO, 2016). The policy recommendation to *test-and-treat* follows a long history of treatment guideline alterations as CD4 count eligibility for ART was expanded from an initial threshold of ≤200 cells/mm^3^ to ≤350 cells/mm^3^ in 2010 and ≤ 500 cells/mm^3^ in 2013 (WHO, 2015, 2016). The latest change in guidelines was accompanied by calls for “rapid adoption” of the changes into national policies (WHO, 2015) in order to meet the treatment targets set by UNAIDS - an almost doubling of the number of people receiving ART from an estimated 18.2 million in mid-2016 to about 30 million in 2020. To achieve this, the treatment targets (also known as the 90-90-90) stipulate that 90% of all people living with HIV need to know their status; 90% of all people with diagnosed HIV infection need to receive sustained antiretroviral therapy; and 90% of all people receiving antiretroviral therapy need to achieve viral suppression by 2020. The ‘rapid’ adoption of *test-and-treat*, is seen as a critical step in enabling a supportive policy and implementation environment to achieve these global treatment targets (Church et al., 2015). Gupta and Granich (2016) argue “there is an urgent need to shorten the time lag in adopting and implementing the new WHO guidelines recommending ‘treatment for all’ to achieve the 90-90-90 targets”.

A review of studies reporting on health policy adoption in low- and middle-income countries highlights the absence of temporality in such studies (Gilson and Raphaely, 2008). Instead, much of the empirical literature emphasises structural explanations; making explicit criterion for effectiveness and success; and considering the policy process as evolving through a sequence of discrete stages or phases (Ambia et al., 2017; Cawley et al., 2017; Gilson and Raphaely, 2008; McRobie et al., 2017; Tlhajoane et al., 2018). Although such frameworks are valuable for highlighting areas for improving service delivery from a resource and health system perspective, such approaches elicit exploration of the impact and effects of implementation, and the perceived ‘success’ or ‘failure’, at the expense of focus on the processes, movements and temporality of the policies. It is against this background that we ask: What slows down or speeds up the practices of adopting and implementing the HIV *test-and-treat* policy?

## 2 Methods

This study forms part of a larger mixed-methods policy study that sought to evaluate the health system in Zimbabwe concerning its implementation of HIV policies in the advent of universal eligibility of ART. The study was approved by the Medical Research Council of Zimbabwe (MRCZ/A/2151) and Imperial College London's Research Ethics Committee (16IC3597). Informed and written consent was obtained from all participants with the agreement that confidentiality was upheld. We have used pseudonyms throughout.

### 2.1 Study location and participants

Generally, Zimbabwe has made good progress in implementing international guidance on HIV service delivery (Gregson et al., 2017; Tlhajoane et al., 2018). Strategies have been developed and adapted to fit the local context. These include the decentralisation of HIV care and treatment services to ensure universal accessibility to ART (began in June 2016 with some pilot sites, and nationally in December 2016), the introduction of Option B+ (immediate, lifelong ART for all pregnant and breastfeeding women with HIV), and task-shifting of HIV care from medical doctors to non-physician clinicians (Tlhajoane et al., 2018).

Participants were recruited from two settings. Seven national policymakers were recruited in Harare and nine sub-national policymakers were recruited from the eastern province of Manicaland. Participants were purposefully recruited to represent a variety of stakeholders involved in the adoption of the 2015 WHO guidelines and *test-and-treat* implementation. National policymakers included representatives from the Ministry of Health, the National AIDS Council and National Medicines and Therapeutics Policy and advisory Committee (NMTPC) as well as major programme implementers, such as Organization for Public Health Interventions and Development (OPHID), FHi360, Population Services International (PSI), Elizabeth Glaser Pediatric AIDS Foundation (EGPAF). Provincial stakeholders included provincial HIV programme coordinators and senior health facility workers from three rural administrative districts in Manicaland Province (Makoni, Mutasa and Nyanga). Health facility workers from the three districts were recruited to represent different characteristics of service providers available within each district, including a balance of low- and medium-volume sites, health facility type (e.g., hospital, health centre or rural small clinic), location (e.g., small town, growth point, roadside business centre, rural location) and health facility management authority (e.g., Ministry of Health, private clinics, Christian mission).

### 2.2 Data collection and analysis

This study reports on data from sixteen in-depth interviews. The interviews were conducted using semi-structured topic guides, developed to identify factors that are key determinates of policy implementation. National policymakers were encouraged to reflect on the changing HIV policy environment, how the *test-and-treat* policy was incorporated into national treatment guidelines and the processes of introducing and implementing *test-and-treat* to health facilities. Interviews with subnational policymakers covered themes such as learning about *test-and-treat*; health facility implementation of *test-and-treat*, and the effects on *test-and-treat*. Interviews were carried out in October of 2017, conducted in either English and/or Shona, the local language, in organisation offices or private rooms at health facilities. Interviews were audio-recorded and lasted between forty-five to 90 min. All transcripts were anonymised, transcribed and translated into English before being imported to NVivo 10 for coding. Data was coded following Attride-Stirling's (2001) method of thematic network analysis; to cluster codes into basic and organising themes, to form the development of a broad analytical coding framework. Emerging findings were coded based on an inductive (Mason, 2002) and interpretive approach (Yanow, 1999); deductively clustering inductively-generated codes by analysing the codes in respect of our temporal interest and critical perspective. This process generated twenty-five basic themes, which were grouped together into nine organising themes, which in turn were clustered into three global themes (see Table 1). This network of themes forms the structure of our presentation of results.

**Table 1:** Thematic network of emerging findings.

Emerging themes [basic themes]	Temporalities affecting test-and-treat policy adoption [organising themes]	Core temporalities [global themes]
*-Test-and-treat* was rapidly implemented	Speed	Tempo
- Technologies speed up dissemination		
- Managing the ‘right’ speed		
- Global events were important advocacy initiatives	Momentum	
- National reputation and global recognition were motivating		
- Donor supported services accelerate roll-out	Acceleration	
- Technologies provide new avenues for communication		
- Multiple supportive documents accelerated implementation		
- Working to avail physical guidelines causes delays		
- Perceived natural sequence for how things should be among national policymakers	Sequence	Rhythms
- Variations in perceptions of sequence effected conceptualisation of success		
- Policy changes happen too frequently	Frequency	
- Frequency of change causes mixed-messages for providers and patients		
- Full implementation is harder to evaluate		
- Keeping up with changes is resource intensive		
- Release must match strategic planning	Cyclicality	
- Commodities in stock have to be used before switching		
*-Test-and-treat* was long-awaited	Timeliness	Epoch
- It was a natural succession from earlier policies		
- It came at a time of relative economic stability		
- Experience of Option B+ enhanced policymakers competencies	Histories	
- Previous guideline adaption enhanced know-how		
- Past messaging stuck in the minds of people		
- Low complexity of intervention enhances readiness	Readiness	
- Pilot testing increases readiness for scale-up		

## 3 Findings

### 3.1 Factors affecting the speed, momentum and acceleration (tempo) of *test-and-treat*

International recognition for speedy adoption of *test-and-treat* helped to generate strong buy-in particularly among national policymakers. In July 2017, representatives from Zimbabwe's Ministry of Health were invited to present their experiences of *test-and-treat* at the 9th IAS Conference on HIV Science in Paris. National policymakers reported that Zimbabwe was recognised internationally for the timeliness with which they were able to adapt the WHO recommendations into national guidelines and for being “quite innovative in how they moved to operationalise the guidelines” (R5). The speed at which Zimbabwe was able to carry-out these activities was recognised by many national policymakers:

“When we were rolling out, we were moving very quickly because we now have the guidelines and we wanted to be, you know, ahead of the curve” - (R2)

The recognition afforded by the international community not only amplified the perception of ‘being ahead of the curve’, but also reinforced the momentum already created. While the policymakers found pride in moving at great speed, some also articulated a related tension, namely the need to manage the ‘right’ speed and respect the time it takes to adopt and implement a new policy:

“I think respecting it is a process is important; it does take time. It has to happen in the time it takes, because if you move too fast and you leave segments, stakeholders and people behind, then in the long run it will catch you up. I think some would say actually Zimbabwe moved very quickly.” (R1)

Global advocacy events provided opportunities for information to travel, across the globe. Policymakers in Zimbabwe harnessed the momentum generated at these various scales; as the events and activities seem to act as symbolic assurance of the Government's commitment for *test-and-treat.* According to Gupta and Granich (2016) Zimbabwe was faster at adopting previous WHO recommendations than many of its neighbouring countries and particularly compared to other countries in sub-Saharan Africa. Their data shows Zimbabwe has historically been quick to update their guidelines. Zimbabwe was also quick to adopt the 2015 guidelines, accelerated by their hosting of the 2015 International Conference on AIDS and STIs in Africa (ICASA). At the conference, Zimbabwe announced a commitment to adapt their national guidelines in line with 2015 WHO recommendations and key donors were there “to commit their support to supporting treat all … there was financial backing” (R5). Financial backing from donors accelerated the tempo at which *test-and-treat* could be adopted and implemented; particularly with support from PEPFAR. PEPFAR worked with implementing partners from NGOs and government agencies to help disseminate and execute the guidelines at facility level. Districts where PEPFAR was not working relied on other sources of support and faced “gaps in resources” (R1) to execute the guidelines. These “gaps” affected the tempo at which *test-and-treat* could be rolled-out nationally:

“… the partners are the ones that started earlier, because they had support from [their own] governments. For those that didn't have direct support from partners in terms of distribution, they started quite late” - (R1)

A year after the ICASA conference, on World AIDS Day in December 2016, Zimbabwe officially ‘released’ the national guidelines. It appears the international community have recognised these achievements, which was a source of pride for many national level policymakers: “their desire to learn from us is also something I feel quite proud about: that Zimbabwe is leading in the direction around ‘treat all’” (R5).

Presenting these physical guidelines on World AIDS Day was a significant marker of successful implementation for many national policymakers. However, working to avail the physical copies of the guidelines was resource intensive and required the Ministry to collaborate with multiple partners; ultimately slowing down the tempo at which printed copies could be “on the desks” of health facility implementers.

“If we had enough resources, we could have printed the documents earlier; disseminating and distributed them. We need to have an effective way of distributing our materials because we are waiting … we are riding on the drug distribution system of NatPharm and we noticed delays in terms of getting these guidelines out” - (R1)

Focusing on the timely distribution of the physical copies to the health facilities as a key indicator the success of “rapid” implementation, can overlook the value of ‘soft-copies’ and alternative versions of the guidelines to speed-up policy adoption and implementation. The development of an Operational and Service Delivery Manuel (OSDM) and updated ‘Job Aid’ across all HIV/AIDS and STI programmes, supported the rapid implementation of *test-and-treat* on two main counts: first, these documents were more widely and quickly distributed (by e-mail or WhatsApp) compared to the national guidelines, thereby circumventing the delays of the national guidelines to health facilities: secondly, they provided practical information for implementers on how to roll-out *test-and-treat*. Alongside the OSDM and Job Aid, many NGOs also made their *own* forms of guidelines: Standard Operating Procedures (SOPs); Monitoring and Evaluation (M&E) tools. They were used as a way of translating and transporting messages from national level to subnational level; particularly for those left out of decision-making and training activities. The OSDMs and Job Aids were a way of overcoming these information barriers and hierarchies, by being a means to communicate new messages with the authority of the Ministry. All these documents together created momentum for the implementation of *test-and-treat* despite the delays to official guideline distribution.

These alternate modes of “getting to know” the guidelines enabled information to be shared quickly and unofficially. One national policymaker was concerned, however, that the “quick flow of information” undermined the accuracy of the information within the guidelines because information could be shared before being formally “signed off” (R4). To avoid the “risk” of “things happening so fast” and electronic versions going “out when they are not quite ready to be sent out”, the respondent suggests “it will be nice to wait to see a print copy; then we know we've set the processes over” (R4). For the Ministry, the timely distribution of the physical copies to the health facilities was a key indicator the success of “rapid” implementation, perhaps with little avail; because “once the guidelines were out the majority of the districts had already transitioned” (R5), that is, they had received the guidelines in various other forms and means.

### 3.2 Experiences of sequence, frequency, and cyclicality (rhythm) affecting *test-and-treat*

There were three broad ways policymakers (at both national and subnational level) conceptualised the ‘processes’ of *test-and-treat* implementation: one, “localising” the guidelines; two, “getting to know” the guidelines; and three, “operationalising” the guidelines. The process of “localising” the guidelines was also referred to as “adapting” and “adaptation at country level” (R2), that is, adapting the WHO recommendations so they are better suited the Zimbabwean context. There were several activities associated with this process: learning districts; stakeholder meetings; technical meetings and workshops; updating materials. National policymakers spoke positively of the adaption process, often describing the activities as collaborative, efficient and well-led – “within a period of three months, this is done: the guidance is now localised and internalised” (R2). According to many narratives, a “sensitisation” processes of “getting to know” the guidelines followed the national adaption phase. From the Ministry's account, this involved a dissemination meeting two-weeks prior to the launch and a three-day workshop to disseminate all the guidelines. From there, “subnational leadership are expected to cascade the information to the health facilities” (R1). The following process of “implementation” or “operationalising” the guidelines (R5) was where many of the national policymakers identified the “main challenge” to implementation of *test-and-treat*.

A successful strategy for early *test-and-treat* adoption and implementation was running a pilot project, led by NGOs. These pilot projects were considered “learning districts” – a way of generating early discussions, learning and feedback opportunities for national *test-and-treat* roll-out. Beginning some activities early and running multiple activities in parallel, helped to increase the speed at which *test-and-treat* could be adopted:

“We started doing treat all learning districts in April, May, June and then it was that August they were talking about the ART guidelines. So, you see in some districts treat all was already rolling out as well when some of the stakeholders were now talking about the guidelines and that meant we also had some evidence to be able to share” - (R5)

Implementing *test-and-treat* at different times helped overcome delays at national level:

“One of the challenges I had mentioned at national level was that sometimes the processes are long, and it takes a while from recommendation to implementation, but I think we [NGO] didn't quite have that challenge. We started treat all way before the national system had rolled it out” - (R6)

Some policymakers were more explicit in how they perceived a “correct” sequencing of events, by referring to the circumstances in which perceived order and control was lost and the phased and linear approach was undermined. Others also noted how keeping up with the frequency of changes to HIV treatment policies, drugs and technologies required more work and resources for national policymakers, slowing down time to fully implement and see the results, as one policymaker commented:

“Some of the challenges are the frequencies of changes in the guidelines because we only launched the guidelines last year. It's not even a year and already there are changes to the global guidelines and we are expected to again go through this process. It doesn't give us space to implement” - (R1)

Having to undo old messages “stuck in the minds of people” presented extra challenges for subnational policymakers in the counselling period for patients (R13), ultimately complicating and slowing down the counselling and patient's acceptance stage. This undermined the full implementation of the *test-and-treat* policy, which specifies that a newly diagnosed patient is required to initiate ART *within seven days* after being tested positive. The patient must have “accepted” their diagnosis and be ready to start initiation, after adequate guidance and counselling from the provider. Before the 2015 recommendation, previous practice was to provide three to four pre-ART counselling sessions until the patient was deemed ready to start ART (ZMOHCC, 2014). Many subnational policy implementers expressed concern at the short limit for linkage:

“The only fear that I have is on the time limit for linkage. I think it's a bit small, that we need maybe to say two weeks, then someone would have understood … because the seven-day limit is a very short time for a person to make a very concrete decision” - (R10)

As some health facility workers pointed out, automatically moving to the new guidelines was not possible due to the timing of the release of the guidelines, which did not align with their strategic planning. When *test-and-treat* policy was introduced the Zimbabwe National HIV and AIDS Strategic Plan (ZNASP), which stretched from 2011 to 2015, was ending, so too were the WHO directed targets ’15 by 15’.^1^ The mismatch between national strategic planning, the 90-90-90 WHO targets, the 2015 WHO guidelines, the Zimbabwe 2016 national guidelines and the new ZNASP from 2015 to 2018, was said to slow down the process of *test-and-treat* adoption. Similarly, the new guidelines had to match the cycles of commodity procurement: commodities in stock had to be used before switching to new recommendations, causing delays.

### 3.3 The timeliness, histories and readiness (epoch) of *test-and-treat* practices

The common rhetoric among all policymakers was that *test-and-treat* was long awaited and timely, as it made sense scientifically and in practice. National policymakers involved in adapting the WHO recommendations into national guidelines, often referred to the strength of the scientific evidence as a motivating factor for “easy” acceptance and “a lot of consensus” among policymakers because “it made a lot of sense in the medical field, rather than waiting for someone to be sick, to put them on medicine” (R1). By addressing issues of loss to follow-up, *test-and-treat* was seen as helping Zimbabwe reach the 90-90-90 global treatment targets:

“People were already of the mood that okay if we have already accepted everybody else, why do we make these other ones wait? Because we may lose them. Loss to follow up was a big concern and for all the people that you test positive and then tell them that they are not yet eligible for treatment, do they come back?” (R5)

*Test-and-treat* came as a “relief” for subnational health facility staff because it meant they did not have to deny patients treatment based on the previous eligibility criteria; avoiding confrontation and uncomfortable justification for why some patients were eligible for treatment, while others were not:

“It came as a relief to us because we were having challenges with some of the clients that we were helping in the community that were not getting access to the OI [Opportunistic Infections] clinic because of their clinical condition. They were being delayed because of the eligibility criteria. With *test-and-treat*, everyone was eligible and that was a relief to the clients as well as to the organisation” - (R10)

Many subnational policymakers were motivated by evidence of *test-and-treat* working *in practice*. Being the end users of *test-and-treat* and having contact with patients meant that subnational policymakers “observed that it worked” (R13). They were able to see how the implementation “quickly improved a person's life” because they could see how immediate testing and treating “reduced the burden for follow-up visits … and also reduces morbidity” (R10).

Although the frequency of changes to guidelines caused confusion and mixed-messages on one hand, the process of adapting the 2015 WHO guidelines to national guidelines was simplified and sped-up *because* of the experience gained from adapting, and implementing, previous recommendations from the WHO. One respondent said that adapting the guidelines to include *test-and-treat* was “an easy process because we've gone through three or four launches of HIV adaption: 2004, 2006, 2010, 2013, 2015, so we draw lessons from those guideline adaptions” (R7). Roll-out of the 2013 WHO recommendation (Option B+), gave implementers practical know-how and operational tools to support rapid adaption and roll-out of *test-and-treat*. Adopting Option B+ to national guidelines in Zimbabwe was quick, taking only six months (Gupta and Granich, 2016), as was the speed at which sites were offering the policy across the country: “we transitioned to eighty-five percent of sites within a year and then in the next quarter we were able to get ninety-five percent” (R2). Many national level respondents consequently mark the transition from the previous regimen (prophylaxis) to Option B+ as the start of the transition toward *test-and-treat*. One respondent said that “Option B+ *was test-and-treat*” (R4). In this view, since *test-and-treat* was an extension of Option B+, roll-out required minimal staff training or adjustment to practice. It was generally reported among subnational policymakers that implementing *test-and-treat* did not significantly change the workload for staff, nor require more resources. In fact, it was commonly reported that “there was not much change” (R9) with *test-and-treat,* only a change in eligibility criteria – “what we just have done is shorten the period of time in terms of initiation” (R11).

## 4 Discussion

Our findings shed light on some of the ways in which different tempos, rhythmic experiences and epochal practices come together to shape the speeding up and slowing down of *test-and-treat* implementation in Zimbabwe. Findings suggest there to be multiple and parallel activities leading to “rapid” adoption and implementation, which circumvents the phased and linear approach often portrayed in policy transfer and implementation literature. We found these factors, experiences and practices to be highly temporal and contingent on spatiality and materiality in specific and unique contexts. We also found this diversity of factors to work in synergy and through a series of relational connections, rather than silos and separations.

Our findings point to a broad range of factors, experiences and practices affecting the tempo of *test-and-treat* roll-out. Global conferences, seminars and workshops accelerated the development of a politically willing environment for *test-and-treat* in Zimbabwe. At these high-profile events key stakeholders were brought together, facilitating the acceptance for, and momentum of, rapid adoption and implementation of *test-and-treat*. Other policy mobility scholars have drawn attention to how conferences, seminars and workshops are fundamentally political in nature because they are places where persuasion and negotiation occur. McCann and Ward (2013:13) argue “they are situations in which formal and institutional decision-making processes occur … they are locations for inter-personal persuasive politics”. In line with a geographical perspective, our results find that these sites were indeed political and also referential in that “it always involved stories and evidence of, and about, ‘elsewhere’” (McCann and Ward, 2013:11). The events also supported processes “saturated by power relations” (Clarke et al., 2015; Fischer et al., 2015; Peck, 2011); as being left out of meetings, trainings and sites of decision-making contributed to slower acceptance for *test-and-treat* and more resistance from subnational policymakers. We also noted that the implementation of *test-and-treat* was discursively constituted; wrapped up in narratives of being “ahead of the curve” and “leading the way” – affecting, and being affected by, the momentum that was generated around *test-and-treat* in Zimbabwe. These and other messages “filtered down” from the national level to subnational level, demonstrating the idea that policy regimes are “relationally interconnected” (Peck et al., 2012: 273) and “local politics is global” in the context of best-practice models being transferred from one context to the next (Campbell et al., 2012a; Temenos and McCann, 2013). The material manifestation (or lack thereof) of *test-and-treat* also affected the tempo at which *test-and-treat* was implemented. Although the process of “localising” the guidelines was considered timely and efficient, the dissemination of a physical copy of the guidelines was described as delayed, slow and resource intensive. The time and resources used to disseminate the physical guidelines turned out to have little value for a ‘rapid implementation’ perspective. Instead, a number of official and unofficial supportive documents quickly got into circulation, accelerating the tempo of *test-and-treat* roll-out. In the time between adapting 2015 WHO recommendation to national guidelines, various national policymakers from NGOs created their own forms of guidelines, in the form of SOPs and M&E frameworks, to support early activities associated with *test-and-treat*. The Ministry also developed other official supportive documents, like the Job Aid and OSDM. These multiple forms of “guidelines” could be seen as a literal form of policy mutation (Peck, 2011). These different forms, in hard and soft copies, were faxed, e-mailed and shared through social media platforms, providing national and subnational policymakers with programming tools for early *test-and-treat* implementation. In other words, *test-and-treat* travelled in various forms, through various networks and sites, at various tempi (Temenos and McCann, 2013).

*Test-and-treat* also had to fit into a number of rhythmic activities, shaping the experiences of policymakers. Some national policymakers alluded to the challenges of “a very rapid scale up” as disturbing the perceived natural rhythm and sequence of policy adoption and implementation. Mismatches were also noted between the planned cycles of global target timeframes and national strategic planning periods, as well as drug procurement planning and usage. In light of these rhythmic tensions, a number of national policymakers alluded to the benefits of not rushing to implement and to respect the time it takes to roll-out *test-and-treat.*

We found *test-and-treat* to be epochal in nature by being historically timely. It introduced a long-awaited change in HIV treatment policy that built upon past events and practices. The fact that *test-and-treat* was a development of Option B+ from the 2013 WHO recommendations gave policymakers practical knowledge and know-how for smoother and more rapid implementation of *test-and-treat*. Similar observations have been made by McRobie et al. (2017), who noted that Option B+ was an important part of the planning process for policy implementation in Uganda, where piloting and learning from past experiences allowed for revision of practices for scale-up. In Zimbabwe we found that *test-and-treat* “made-sense” to policymakers because, in the context of Option B+, it represented “just” a change in eligibility criteria. Policymakers had gained experience and competencies in how to develop, manage and execute the adaption and implementation processes, and many of the health system structures for the drugs supply and HR were there, they just needed to be strengthened.

### 4.1 Towards a temporal framework for policy adoption analysis and action

Our study has identified numerous temporalities that both affect, and are affected by, the policy adoption practices of policymakers. It is in the language of how they describe *test-and-treat* implementation (“quick to implement”, “rapid”, “fast”, “moving quickly”). There are references to the past, present and expectations for the future. There are references to the effect of time needed to move through space on the logistics of implementing *test-and-treat* and the specific time periods required from the policies themselves (seven-day time limit for initiation; 90-90-90 targets period until 2020). There are other more implicit temporal conceptualisations by policymakers, in referring to good timing, experience over time and time needed to change and bring acceptance. These temporal manifestations become amplified in the context of the global call for “rapid” adoption and implementation of such policies.

Given the strong presence of temporality in our findings, we take inspiration from Cresswell's (2010) “constellations of mobility” and conceptualisation of policy movement and draw on our findings to propose an analytical framework for studying the ‘temporalities of policymaking’. Our findings highlighted three main temporal themes, each with three temporalities, which can structure such an analysis (see Table 2). These temporalities, aided by our analytical questions, can be used as a lens to foreground the temporal aspects of policymaking. It is important to appreciate that none of these temporalities operate in isolation: they are mutually implicating and forming. Nor are these temporal typologies intended to be exhaustive: they are likely to differ from context to context and in time and place. As such, these temporalities of policymaking are intended to initiate what Peck (2011: 774) refers to as “a rolling conversation, rather than a coherent paradigm.”

**Table 2:** Analytical framework for studying the temporalities of policymaking.

Tempo			Rhythm			Epoch		
*Speed*	*Momentum*	*Acceleration*	*Sequence*	*Frequency*	*Cyclicality*	*Timeliness*	*Histories*	*Readiness*
*How fast a policy moves or is put into action*	*The force that keeps the policy moving, after it has been introduced*	*Increase in the speed at which a policy is implemented*	*The order in which policy-related events or practices follow each other*	*The number of times policy-related events happen within a particular length of time*	*A series of policy-related events or practices that happen in a particular order, and are often repeated*	*Policy and related practices introduced at the best or worst possible time*	*Past policy-related events and practices considered together*	*Willingness or a state of being prepared for the policy*

How fast does a policy move? Does everyone experience or conceptualise speed in the same way? When do things speed up, when do things slow down? What are the effects of speed (ing)?	What helps sustain momentum? What helps generate buy-in?	What makes things move faster? What makes things slow down?	Is there a perceived order or series of related events? Do these perceptions vary between different people, why, to what effect?	How often do policy changes take place, why, to what effect?	Do timings make sense? How does the policy and related events fit with other policy-related practices? What are the effects of time needed to move through the practicalities?	Does it make sense in the wider context of past, present, future expectations? Is it a good time for action? What is the social, political, economic, environmental, religious context of timing?	What competencies have been developed from past experiences? What is the role of memory and habit? What routines have been made, by who, to what effect?	Is the policy introduced at a ‘good time’ or ‘bad time’? What determines the timeliness of the policy? What supporting structures exist or need to be developed?

Analysing the temporalities of policy roll-out helps uncover how the process of implementation happens unevenly in contested and contradictory ways, rather than being determined via a hierarchical, linear and rational process. Policies are made sense of in their own context, which have their own histories, geographies, and policy innovations, preferred models and best practices. By contextualising the implementation process through a temporal lens, we see that implementing policy does not happen in isolation but is affected by past experiences, present practices and future expectations of for instance, the sustainability of *test-and-treat*. By making the complex processes of policy implementation transparent, policymakers and policy analysts are better able to critically assess the concrete, local and experienced effects of national policies.

The momentum at which scientific evidence, evidence-based policy, new technologies and innovative actions are being produced and circulated is unlikely to slow down; and nor should it. But, it is in this context, supportive environments for “rapid” policy adoption are imperative, as is understanding *how* “rapid” adoption and implementation might be possible. By unpacking the temporalities of policy adoption processes, we have shown how three thematic temporalities (tempo, rhythm, epoch) can help shed light on the factors, experiences and practices that affected rapid adoption and implementation of *test-and-treat* policy in Zimbabwe. Exploring these constitutive parts, singly and together, has enabled an understanding of “successful” implementation of *test-and-treat* beyond standard health system performance measurements. Our findings suggest that exploring health system factors without regard to temporality may miss some important explanations as to *how* a policy is rolled out. Assessing the rate at which countries adopt and subsequently implement policies and treatment guidelines is therefore not as straightforward as simply measuring the date at which national guidelines are released.

The empirical findings presented above need to be considered in the context of a few limitations. The study was conducted in two predominately rural districts in Manicaland, which is currently the province with the lowest HIV prevalence (ZMOHCC, 2017). The facilities may not be representative of facility performance across Zimbabwe, as routine research activities associated with the Manicaland Project may have benefited service delivery. The fact that certain districts of Manicaland Province were selected for the pilot study of *test-and-treat* may also have influenced the responses of policymakers at subnational level. However, the potential ‘newness’ of the processes involved in policy implementation also offers interesting perspectives relevant to the focus of this study. Response bias is also a potential limitation to the study. Many of the respondents *qua* their role as policymakers and implementers may have had a vested interest in portraying things as having been successful and playing down the problems. Moreover, our findings lack a local user and communities' perspective. Future research may adopt a more ethnographic approach to mitigate some of the aforementioned limitations.

By and large, *test-and-treat* in Zimbabwe has been successfully adopted and implemented – in “rapid” time. In just over a year, the policy to provide *treatment for all* was adapted and adopted into national policy and all new, known and willing, patients had started HIV treatment. As a result, approximately 87% of Zimbabweans aged 15–64 who have been diagnosed with HIV (74.2%) are on antiretroviral treatment (ZMOHCC, 2017). This research has explored and outlined some of the place-specific factors, experiences and practices that converge to shape the “rapid” adoption and implementation of *test-and-treat* in Zimbabwe. In identifying nine temporal constituent parts to “rapid” adoption and implementation of *test-and-treat*, we not only show policymaking to be a complex, messy and temporal affair, but present a temporal lens for policy adoption analysis that could support future calls for “rapid” policy adoption and implementation.

## Footnotes

1 In March 2015, the goal of reaching 15 million people with HIV treatment by 2015 (also known as '15 by 15') was achieved ahead of the deadline (Gupta and Granich, 2016).
